# Ocular hypotensive effect of fixed-combination brinzolamide/brimonidine adjunctive to a prostaglandin analog: a randomized clinical trial

**DOI:** 10.1038/eye.2016.126

**Published:** 2016-07-01

**Authors:** R D Fechtner, J S Myers, D A Hubatsch, D L Budenz, H B DuBiner

**Affiliations:** 1 Department of Ophthalmology, SUNY Upstate Medical University, Syracuse, NY, USA; 2 Wills Eye Hospital, Philadelphia, PA, USA; 3 Alcon Laboratories, Inc., Fort Worth, TX, USA; 4 Department of Ophthalmology, University of North Carolina, Chapel Hill, NC, USA; 5 Clayton Eye Center, Morrow, GA, USA

## Abstract

**Purpose:**

To determine whether intraocular pressure (IOP) lowering with fixed-combination brinzolamide/brimonidine (BBFC) adjunctive to a prostaglandin analog (PGA) was superior to that of vehicle+PGA in patients with open-angle glaucoma or ocular hypertension who were inadequately controlled with PGA monotherapy

**Methods:**

This 6-week, multicenter, randomized, double-masked, parallel-group trial was conducted at 30 clinical sites in the United States between October 2013 and May 2014. Eligible patients were adults with open-angle glaucoma or ocular hypertension and with mean IOP ≥21 and <32 mm Hg, whereas receiving an open-label PGA (latanoprost, bimatoprost, or travoprost). Patients instilled a PGA once-daily in a run-in phase before randomization to masked BBFC or vehicle adjunctive treatment. Masked treatments were instilled 3 times daily for 6 weeks, and patients continued once-daily use of their PGA. The primary efficacy end point was the between-group difference in mean diurnal IOP (average of 0800, 1000, 1500, and 1700 hours time points) at week 6.

**Results:**

At week 6, mean diurnal IOP with BBFC+PGA was lower than with vehicle+PGA (17.1±0.4 mm Hg *vs* 20.5±0.4 mm Hg; between-group difference, −3.4±0.5 mm Hg; *P*<0.0001; 95% confidence interval, −4.5 to −2.4 mm Hg). BBFC+PGA reduced mean diurnal IOP by 5.7 mm Hg (25%) from the baseline IOP achieved with PGA monotherapy.

**Conclusions:**

Therapy with BBFC produced an additive IOP-lowering effect compared with a PGA alone or in conjunction with vehicle. BBFC may provide an effective treatment option for patients receiving PGA monotherapy who require additional IOP reduction.

## Introduction

Insufficient intraocular pressure (IOP) control is a primary risk factor for progression of ocular hypertension and glaucoma,^1^ and elevated IOP is associated with increased risk for vision loss.^2^ Prostaglandin analogs (PGAs) are highly effective first-line therapies for lowering IOP.^3^ As many as 50% of patients in the Ocular Hypertension Treatment Study required additional ocular hypotensive medications to maintain IOP reduction after the first year of treatment.^4^ Although the safety and efficacy of adding a fixed-combination medication containing timolol to prostaglandin monotherapy have been demonstrated,^5, 6, 7^ data are needed to evaluate the addition of a fixed combination without a *β*-blocker to PGAs.

Brinzolamide 1%/brimonidine 0.2% fixed-combination ophthalmic suspension (BBFC; SIMBRINZA, Alcon Laboratories, Fort Worth, TX, USA) is currently the only fixed-combination glaucoma therapy that does not contain a *β*-blocker.^8^ In randomized, double-masked trials, treatment with BBFC 2 times daily (BID; approved dosing in most countries) or 3 times daily (approved dosing in the United States) significantly reduced IOP by ~20–35% from baseline, and BBFC was more effective than either of its components.^9, 10, 11, 12, 13^

The objective of this study was to determine whether IOP lowering with BBFC adjunctive to PGA (BBFC+PGA) was superior to that of vehicle+PGA in patients with open-angle glaucoma or ocular hypertension who were inadequately controlled with PGA monotherapy.

## Patients and methods

### Study design, treatment, and randomization

This 6-week, randomized, parallel-group trial was conducted at 30 sites in the United States between October 2013 and May 2014 (ClinicalTrials.gov ID, NCT01937312). The study included 5 visits conducted in 2 phases: phase 1 was an open-label run-in phase in which patients received an investigator-assigned PGA, and phase 2 was a double-masked treatment phase in which patients were randomized to receive BBFC or vehicle in addition to the PGA used during phase 1.

The open-label phase consisted of a screening visit and 2 eligibility visits conducted 3–8 days apart, with the first eligibility visit conducted up to 28±3 days after screening based on the washout schedule of prior medications. At screening, patients discontinued other IOP-lowering medications and were assigned by study investigators a branded PGA: XALATAN (latanoprost 0.005% Pfizer, New York, NY, USA), LUMIGAN (bimatoprost 0.01% Allergan, Irvine, CA, USA), or TRAVATAN Z (travoprost 0.004% Alcon Laboratories) to be taken once-daily at bedtime for the duration of the study. A ratio of ~2 : 2 : 1 was achieved by imposing ceilings on the total numbers of patients receiving each investigator-assigned PGA. Patients who met all inclusion/exclusion criteria at both eligibility visits were randomized to masked BBFC or vehicle 3 times daily at ~0800, 1500, and 2200 hours for 6 weeks in addition to their once-daily PGA. On-therapy follow-up visits were scheduled after 2 weeks±3 days and 6 weeks±5 days of treatment with BBFC+PGA or vehicle+PGA.

For the randomized, double-masked treatment phase of the study, eligible patients were sequentially randomized by designated personnel at each study site to BBFC or vehicle using a schedule provided by the study sponsor. The series of randomization numbers were generated using PROC PLAN in SAS 9.1 (SAS Institute, Cary, NC, USA) to generate 100 balanced blocks of size 4 in a 1 : 1 ratio. Blocks were allocated on demand using an electronic-data-collection system with interactive response technology web software and stratified by investigator and patients' study PGA to ensure a balance of treatment allocations within study sites and type of PGA.

To minimize potential selection bias, patients, investigators, study site personnel, the study sponsor, and clinical monitors were masked to treatment assignments. Multiple study sites were used to eliminate the potential of single-observer bias. Personnel administering study medications during on-therapy visits did not discuss treatments or participate in IOP measurements, and study investigators were not allowed to administer treatments during the study. BBFC and vehicle were provided to study investigators using identical masked bottles, labels, and packaging with the kit number indicated, and the randomization schedule was restricted until after the database was locked and the study was unmasked.

The study was performed in accordance with Good Clinical Practice and the Declaration of Helsinki, and participating patients provided informed consent. The study protocol and consent documents were approved by Western Institutional Review Board (Olympia, WA, USA), Wills Eye Hospital Institutional Review Board (Philadelphia, PA, USA), Summa Health System Institutional Review Board (Akron, OH, USA), and Sterling Institutional Review Board (Atlanta, GA, USA) before the start of the study.

### Patients

Patients were enrolled by individual study investigators. Eligible patients were aged ≥18 years, diagnosed with open-angle glaucoma (including pseudoexfoliation or pigment dispersion) or ocular hypertension, and had a mean IOP of ≥21 and <32 mm Hg in at least 1 eye at 0800 hours during both eligibility visits, whereas on PGA monotherapy. Patients with IOP ≥32 mm Hg in either eye at any time point were excluded.

Additional key exclusion criteria were central corneal thickness >620 *μ*m, Shaffer angle grade <2, cup-to-disc ratio >0.8, or severe central visual field loss in either eye; chronic recurrent or severe inflammatory eye disease; clinically significant or progressive retinal disease; ocular surgery or trauma ≤6 months before screening; ocular laser surgery, infection, or inflammation ≤3 months before screening; best-corrected visual acuity worse than 55 Early Treatment Diabetic Retinopathy Study letters (equivalent to 20/80 Snellen or 0.6 logMAR) in both eyes; and use of additional ocular hypotensive medications during the study.

### End points

The primary efficacy end point was the between-group difference in mean diurnal IOP (average of 0800, 1000, 1500, and 1700 hours measurements) at week 6. Secondary efficacy end points were mean IOP at each time point at week 6, mean diurnal IOP change from baseline at week 6, and percent diurnal IOP change from baseline at week 6. Mean diurnal IOP, mean and percent diurnal IOP change from baseline, and IOP at individual time points at week 2; and mean and percent IOP change from baseline at individual time points at weeks 2 and 6 were also assessed. Mean IOP at each time point at week 6 was considered a supportive end point in the protocol, but was elevated to a secondary end point before completion of the study. This change was noted in the statistical analysis plan before study completion.

IOP was measured by an operator and a reader using Goldmann applanation tonometry. Two measurements were taken for each eye. If measurements differed by ≤4 mm Hg, the average value was considered the mean IOP for that eye. If measurements differed by >4 mm Hg, a third measurement was taken and the two closest values were averaged. If the three measurements differed by equal amounts, all three measurements were averaged. IOP was measured at screening and at 0800 hours (±30 min), 1000 hours (±30 min), 1500 hours (±30 min), and 1700 hours (±30 min) during the two eligibility visits and at the week 2 and week 6 follow-up visits.

Safety evaluations included adverse event (AE) reports, automated perimetry, dilated-eye examination, best-corrected visual acuity, and slit-lamp biomicroscopy. Best-corrected visual acuity and biomicroscopy assessments were performed at the screening, eligibility, and on-therapy follow-up visits. Perimetry and dilated fundus examination were performed at screening and at the week 6 follow-up visit.

### Statistical analyses

Efficacy end points were analyzed in the intent-to-treat data set (all patients who received study medication and completed at least 1 scheduled on-therapy visit). The safety data set included all patients who received study medication.

Data for one eye per patient were used for efficacy analyses. If both eyes were treated, the eye with higher IOP at 0800 hours averaged across both eligibility visits was selected as the study eye. If both eyes had equal IOP at 0800 hours, the eye with higher IOP at 1000 hours was selected. If both eyes had equal IOP at 0800 hours and 1000 hours, the right eye was selected as the study eye. The primary and secondary efficacy end points were analyzed on an observed-case basis using a statistical analysis model that was robust for data that were missing at random. The superiority of BBFC+PGA relative to vehicle+PGA was established if the mean diurnal IOP at week 6 was significantly lower in the BBFC+PGA group *vs* the vehicle+PGA group. Treatment group differences for the primary and secondary efficacy end points were determined using tests based on the least squares means derived from a repeated measures statistical model that accounted for correlated IOP measurements within a patient. A sequential testing approach was used to account for multiplicity of analyses. Patient demographics, including race and baseline characteristics, supportive efficacy end points, and safety data were summarized descriptively. Information regarding race was collected to enable potential *post hoc* analysis of study outcomes by race categories; patients self-identified their race at screening, and no race categories were predefined. All statistical analyses were performed using SAS software (SAS Institute) with *P*<0.05 considered statistically significant.

Assuming a common standard deviation ranging from 3.5 to 3.9 mm Hg for mean IOP and an estimated drop-out rate of 20%, ~200 patients were required to provide ≥90% power to detect a between-group difference in mean diurnal IOP of 2.0 mm Hg using a two-sample, two-sided *t*-test at a significance level of *α*<0.05.

### Trial registration

This study is registered at ClinicalTrials.gov; ID NCT01937312 (https://clinicaltrials.gov).

## Results

### Patients

Of the 282 patients assessed for eligibility, 188 were randomized and received study medication; these patients comprised the safety data set. There were 93 screening failures; most (62.4%) were because patients did not meet IOP criteria at enrollment. The intent-to-treat data set included 182 patients (BBFC+PGA, *n*=88; vehicle+PGA, *n*=94; Supplementary Figure S1). One patient randomized to BBFC+PGA did not receive study medication and was excluded from the intent-to-treat and safety analyses. Most patients completed the study (92.6%, *n*=175/189; Supplementary Figure S1); 11 patients receiving BBFC+PGA and 3 patients receiving vehicle+PGA discontinued. AEs were the most common reason for discontinuation (5.8% overall, *n*=11/189). Discontinuations because of an AE were reported for 10.6 and 1.1% of patients in the BBFC+PGA group and the vehicle+PGA group, respectively.

Most patients in the intent-to-treat data set were female (63.7%), white (63.7%), and diagnosed with open-angle glaucoma (78.6% Table 1). The PGA allocation was similar between groups. Mean diurnal IOP at PGA-treated baseline was 22.7±2.1 mm Hg and 22.4±2.8 mm Hg in the BBFC+PGA group and the vehicle+PGA group, respectively.

### Efficacy

**Figure 1:**
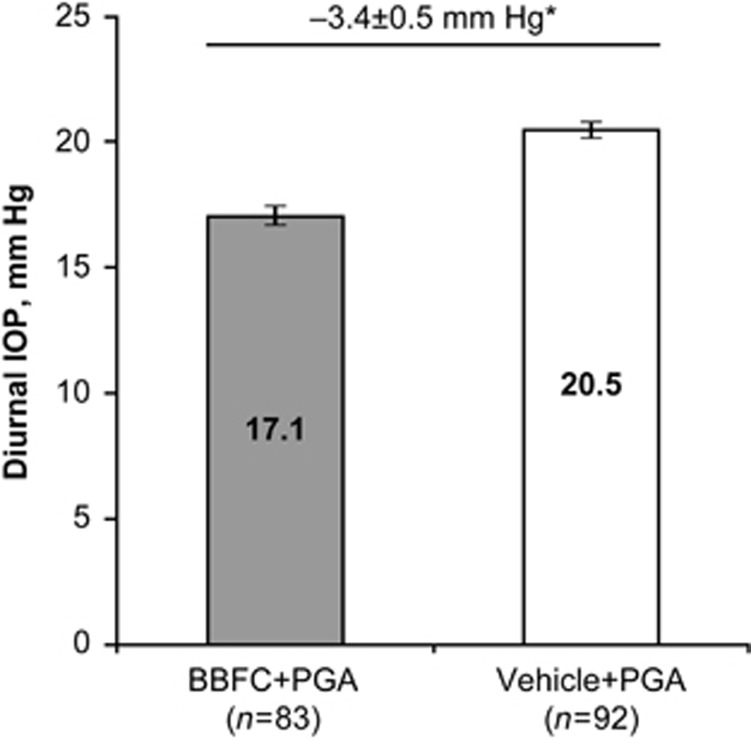
Mean diurnal IOP at week 6. Data are presented as least squares mean and standard error. Between-group difference is depicted above bars. *P<0.0001.

**Figure 2:**
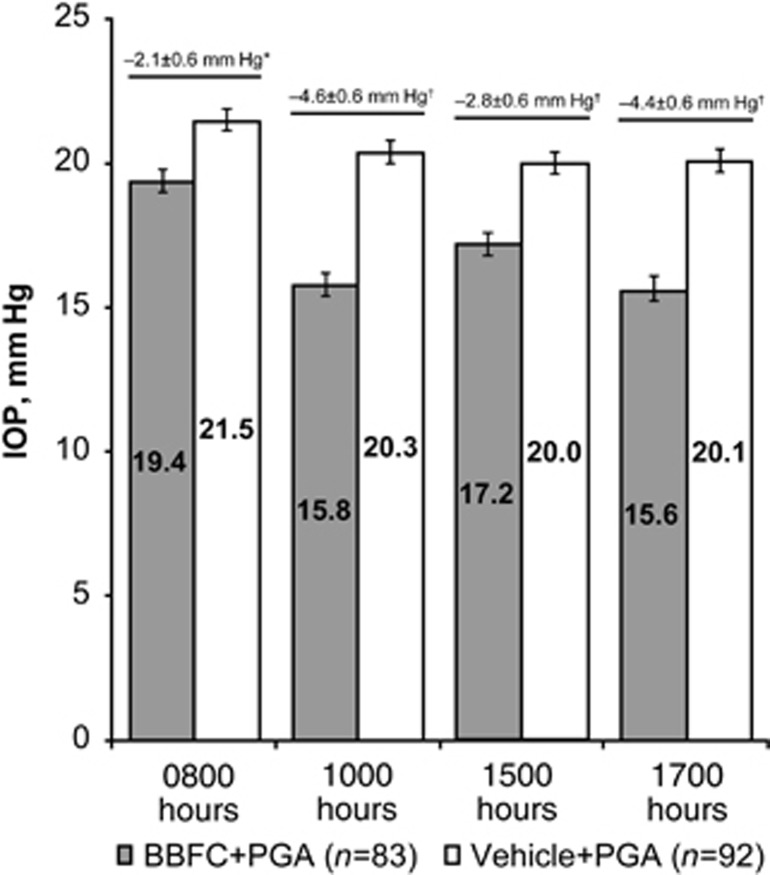
Mean IOP at week 6 time points. Data are presented as least squares mean and standard error. Mean IOP is indicated inside bars; between-group differences are indicated above bars. *P=0.0002; ^†^*P*<0.0001.

**Figure 3:**
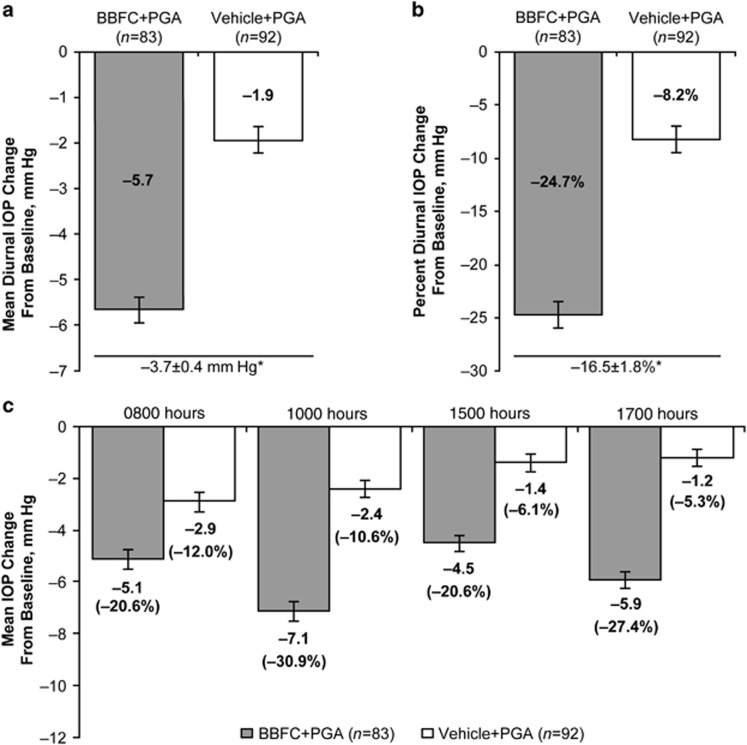
Mean IOP change and percent IOP change from baseline to week 6. (a) Mean diurnal IOP change from baseline; (b) mean percent diurnal IOP change from baseline; and (c) mean IOP change (mean percent change) from baseline by time point. Least squares means and standard error are plotted for diurnal IOP change; descriptive means and standard error are plotted for IOP change at individual time points. Means are indicated within bars; between-group differences are depicted below bars. *P<0.0001.

Mean diurnal IOP at week 6 was significantly lower in patients receiving BBFC+PGA compared with patients receiving vehicle+PGA (Figure 1). Least squares mean±SE diurnal IOP was 17.1±0.4 mm Hg with BBFC+PGA (95% CI, 16.3–17.8 mm Hg) *vs* 20.5±0.4 mm Hg with vehicle+PGA (95% CI, 19.8–21.2 mm Hg). The between-group difference was −3.4±0.5 mm Hg (*P*<0.0001; 95% CI, −4.5 to −2.4 mm Hg); therefore, the criterion for superiority of BBFC+PGA was met. Mean IOP at week 6 was significantly lower at all time points with BBFC+PGA compared with vehicle+PGA (Figure 2; *P*≤0.0002). Least squares mean±SE between-group differences ranged from −2.1±0.6 to −4.6±0.6 mm Hg, with the maximum difference observed at 1000 hours (ie, peak efficacy). Mean diurnal IOP change from PGA-treated baseline was significantly greater with BBFC+PGA (−5.7±0.3 mm Hg) compared with vehicle+PGA (−1.9±0.3 mm Hg). The between-group difference was −3.7±0.4 mm Hg (*P*<0.0001; 95% CI, −4.5 to −2.9 mm Hg; Figure 3a). The percent diurnal IOP change from baseline was also significantly greater in the BBFC+PGA group (−24.7±1.3%) compared with the vehicle+PGA group (−8.2±1.2% between-group difference, −16.5±1.8% *P*<0.0001; 95% CI, −20.0% to −13.0% Figure 3b). Mean IOP change from baseline at each week 6 time point was numerically greater with BBFC+PGA compared with vehicle+PGA (Figure 3c). At the week 6, 1000 hours time point (peak efficacy), the mean±SE IOP change from baseline was −7.1±0.4 mm Hg (−30.9±1.7%) with BBFC+PGA and −2.4±0.3 mm Hg (−10.6±1.5%) with vehicle+PGA. Mean IOP, mean IOP change from baseline, and mean percent IOP change from baseline for diurnal IOP and IOP at individual time points at weeks 2 and 6 are presented in Table 2.

### Safety

The mean±SE duration of exposure to study medication was 39.6±1.1 days and 41.2±0.6 days in the BBFC+PGA and vehicle+PGA groups, respectively. AEs were reported for 35.5 and 21.1% of patients who received BBFC+PGA and vehicle+PGA, respectively (Supplementary Table S1). One patient receiving BBFC+PGA experienced two non-fatal serious AEs (hypoglycemia and metastatic malignant melanoma) unrelated to study treatment. Blurred vision was the most frequently reported AE in both groups and was considered treatment-related in all cases (BBFC+PGA, 9.7% vehicle+PGA, 6.3%). Other common AEs included eye irritation, eye pruritus, and ocular hyperemia; these were reported at higher incidences in the BBFC+PGA group compared with the vehicle+PGA group (Supplementary Table S1). Two severe treatment-related events (pruritus and ocular hyperemia) were reported for the BBFC+PGA group. No serious treatment-related AEs were reported for either group.

Ten patients in the BBFC+PGA group discontinued the study, because of the following treatment-related AEs: allergic conjunctivitis, conjunctival follicles, conjunctivitis, eyelid edema, eye discharge, eye irritation, eye pain, foreign body sensation, increased lacrimation, iridocyclitis, ocular hyperemia, photophobia, pruritus, and blurred vision. One patient receiving vehicle+PGA discontinued because of concussion, joint injury, and skeletal injury. No patients receiving BBFC+PGA and 1 patient receiving vehicle+PGA discontinued the study because of insufficient IOP control.

Minimal changes in visual acuity (<3 letters) were observed in both treatment groups. Ocular signs were generally unchanged from baseline; alterations in conjunctiva and eyelids were more common in patients receiving BBFC+PGA compared with vehicle+PGA (conjunctiva, 12.9 *vs* 4.3% eyelids, 3.2 *vs* 2.1%). Corneal alterations were observed in 3.2% of patients receiving vehicle+PGA and 0% of patients receiving BBFC+PGA. No changes in visual fields or meaningful between-group differences in fundus parameters were observed.

## Discussion

IOP reduction for patients with ocular hypertension or open-angle glaucoma decreases the risk of progressive optic nerve damage and visual field defects. Despite the known efficacy of PGAs, a large retrospective cohort analysis demonstrated that ~12–23% of patients receiving initial monotherapy with bimatoprost, latanoprost, or travoprost required an adjunctive therapy.^14^ Therefore, this study sought to determine whether there would be additional IOP-lowering efficacy if BBFC were added to a PGA in patients not sufficiently controlled with PGA treatment alone.

The IOP-lowering efficacy of BBFC+PGA has not been assessed previously. Our results demonstrated that three-times-daily BBFC produced clinically meaningful IOP reductions when added to a PGA; these reductions were significantly greater than those achieved with vehicle+PGA. A reduction in IOP (diurnal IOP: 1.9 mm Hg, 8% peak reduction: 2.4 mm Hg, 11%) was observed in the vehicle+PGA group, similar to previous observations.^15^ A meta-analysis of clinical trials that assessed common ocular hypotensive agents estimated that vehicle treatment results in small but measurable decreases in IOP ranging from 1.3 mm Hg (5%) at trough to 1.6 mm Hg (5%) at peak.^15^ This effect may reflect regression to the mean or improved compliance with active medication(s) when placebo is added because of participation in a clinical trial; however, consistent observations of IOP reduction with placebo alone suggest that 1 or more components of vehicle formulations may provide a small degree of IOP reduction.

The IOP reductions produced with BBFC+PGA in this study were significantly greater than those reported for brinzolamide+PGA or brimonidine+PGA. A randomized, parallel-group study of PGA-treated patients demonstrated that addition of three-times-daily brimonidine 0.15% or brinzolamide 1% produced IOP reductions from PGA-treated baseline of 4.8 mm Hg (21%) and 3.4 mm Hg (16%), respectively, at 1000 hours (peak effect) after 4 months of adjunctive therapy. In the current study, peak IOP reduction from baseline with BBFC+PGA was 7.1 mm Hg (31%), indicating that adding BBFC to a PGA is more effective than adding brimonidine or brinzolamide individually. This finding is consistent with demonstrations of significantly greater IOP-lowering efficacy of BBFC compared with either component alone.^11, 12, 13^

The effectiveness of treating glaucoma with a fixed combination and a single-agent therapy has been shown previously. After 6 weeks of treatment with fixed-combination brimonidine/timolol added to latanoprost, mean IOP at 1000 hours (peak IOP-lowering efficacy) was reduced by 36% from latanoprost-treated baseline levels of 23.0 mm Hg to 23.4 mm Hg.^5^ This report is not directly comparable to the current study because mean diurnal IOP reductions were not described; however, the 36% IOP reduction at peak was similar to the 31% reduction at the week 6, 1000 hours peak efficacy time point described here. Adding brinzolamide to fixed-combination travoprost/timolol reduced mean diurnal IOP by 14% from the travoprost/timolol-treated baseline of 20.3 mm Hg after 12 weeks.^16^ Further, fixed-combination brinzolamide/timolol or brimonidine/timolol added to travoprost reduced IOP from travoprost-treated baseline (20.1 mm Hg) by 14 and 10%, respectively, after 3 months.^6^ In the current study, BBFC+PGA produced a mean diurnal IOP reduction of 25% at week 6 (compared with 8% in the vehicle+PGA group), suggesting that BBFC added to a PGA may produce IOP reductions similar to those observed in prior studies of a three-agent treatment regimen that included a *β*-blocker.

Previous studies suggest that the magnitudes of IOP reductions achieved with BBFC+PGA in the current study were clinically relevant and may reduce patients' risk of disease progression with long-term use. For example, results of the Early Manifest Glaucoma Trial (EMGT) demonstrated that the risk of progression decreased by 10% with each 1 mm Hg decrease in mean IOP.^17^ In the current study, the mean IOP decrease from baseline in the BBFC+PGA group was ≥4.5 mm Hg at weeks 2 and 6 at all time points assessed. Further, a meta-analysis of 4 studies including a total of 822 patients identified mean IOP as a significant risk factor for progression.^1^ Progression over 5 years of follow-up was reported for more than half of patients with mean IOP>20 mm Hg compared with ≤18% of patients with mean IOP of between 13 and 17 mm Hg.^1^ In the current study, mean diurnal IOP was decreased from 22.7 mm Hg at baseline to ~17 mm Hg at weeks 2 and 6; with the exception of the trough efficacy time point at 0800 hours (19.4 mm Hg), mean IOP at individual time points at weeks 2 and 6 was generally ≤17 mm Hg.

As expected, AEs were more common in patients receiving BBFC+PGA, likely because the BBFC+PGA group was exposed to three active medications compared with only one in the vehicle+PGA group. Furthermore, treatment intolerance or PGA-related AEs likely occurred during the open-label run-in phase of the study. Most treatment-related AEs in the BBFC+PGA group were consistent with the known safety profiles of BBFC, brinzolamide, brimonidine, and PGAs.^8, 18^ No new safety concerns were identified regarding addition of BBFC to a PGA.

A potential limitation of this study was that it did not evaluate the degree to which PGA treatment contributed to IOP control. In addition, because efficacy was evaluated in the eye with higher baseline IOP for all patients (ie, the ‘worse' eye), the absolute magnitudes of IOP reduction observed in the BBFC+PGA and vehicle+PGA groups may be greater than would be observed in a clinical setting (ie, for a patient's ‘better' eye). However, this approach is frequently taken in glaucoma studies to ensure patient safety by monitoring efficacy in the eye at greater risk of glaucoma-related complications and parallels physicians' concerns about managing the more severe manifestation of a patient's glaucoma or ocular hypertension. Because both treatment groups were analyzed using this approach, no treatment bias was introduced.

In conclusion, adding BBFC to PGA therapy produced a mean diurnal IOP reduction of 5.7 mm Hg (25%) compared with PGA-treated baseline and was superior to vehicle+PGA. BBFC+PGA therapy was well-tolerated, and no safety concerns were observed beyond the known safety profiles of the individual agents. PGAs are often preferred as initial monotherapy because of their IOP-lowering efficacy, lower frequency of instillation, and acceptable tolerability profiles. Adjunctive therapy with BBFC can provide an effective option for patients requiring additional IOP reduction beyond that achieved with PGA monotherapy, particularly for patients who cannot tolerate treatment with a *β*-blocker. Future studies incorporating longer treatment durations and comparisons with other fixed-combination medications will provide additional valuable information describing the efficacy of BBFC+PGA adjunctive therapy.


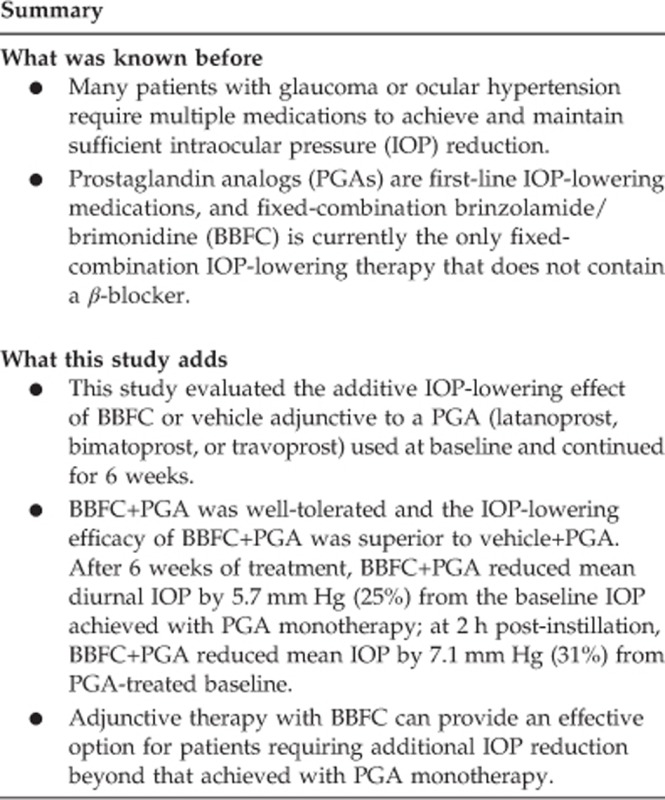


## Figures and Tables

**Table 1:** Patient demographics and baseline characteristics^a^^,^^b^

	*BBFC+PGA (*n=*88)*	*Vehicle+PGA (*n=*94)*
*Age, years*		
Mean±SD	65.5±9.4	64.7±9.6
Range	36–86	30–87

*Sex*, n *(%)*		
Female	53 (60.2)	63 (67.0)
Male	35 (39.8)	31 (33.0)

*Race*, n *(%)*		
White	61 (69.3)	55 (58.5)
Black or African American	25 (28.4)	39 (41.5)
Asian	2 (2.3)	0

*Diagnosis*, n *(%)*		
Open-angle glaucoma	72 (81.8)	71 (75.5)
Ocular hypertension	15 (17.0)	19 (20.2)
Open-angle glaucoma with pseudoexfoliation	0	1 (1.1)
Open-angle glaucoma with pigment dispersion	1 (1.1)	3 (3.2)

*PGA monotherapy*, n *(%)*		
Latanoprost 0.005%	33 (37.5)	37 (39.4)
Bimatoprost 0.01%	31 (35.2)	33 (35.1)
Travoprost 0.004%	24 (27.3)	24 (25.5)

*Diurnal intraocular pressure, mm Hg*		
Mean±SD	22.7±2.1	22.4±2.8
Range	18.0–27.8	16.0–30.5

Abbreviations: BBFC, fixed-combination brinzolamide 1%/brimonidine 0.2% PGA, prostaglandin analog; SD, standard deviation.

a Percentages may not add up to 100 because of rounding off.

b No meaningful differences were observed between treatment groups for any demographic characteristic.

**Table 2:** Descriptive statistics for IOP by visit and time point

	*BBFC+PGA*				*Vehicle+PGA*			
	*Mean±SE IOP, mm Hg*				*Mean±SE IOP, mm Hg*			
	n	*IOP*	*IOP change from baseline*	*Percent IOP change from baseline*	n	*IOP*	*IOP change from baseline*	*Percent IOP change from baseline*
*Baseline*								
0800 hours	88	24.5±0.3	—	—	94	24.3±0.3	—	—
1000 hours	88	22.9±0.2	—	—	94	22.6±0.3	—	—
1500 hours	88	21.7±0.3	—	—	94	21.3±0.3	—	—
1700 hours	88	21.6±0.3	—	—	94	21.2±0.4	—	—
Mean diurnal	88	22.7±0.2	—	—	94	22.4±0.3	—	—

*Week 2*								
0800 hours	88	18.8±0.4	−5.6±0.4	−22.8±1.6	94	21.8±0.4	−2.5±0.3	−10.3±1.3
1000 hours	86	15.7±0.4	−7.2±0.4	−31.5±1.6	93	20.2±0.4	−2.4±0.3	−10.5±1.3
1500 hours	86	16.9±0.3	−4.8±0.3	−21.8±1.4	93	19.9±0.4	−1.5±0.4	−6.1±1.6
1700 hours	86	15.3±0.4	−6.3±0.4	−28.8±1.5	92	19.4±0.4	−1.8±0.3	−7.5±1.7
Mean diurnal	88	16.8±0.3	−6.0±0.3	−26.0±1.3	94	20.4±0.4	−2.0±0.3	−8.5±1.2

*Week 6*								
0800 hours	83	19.4±0.4	−5.1±0.4	−20.6±1.5	92	21.4±0.5	−2.9±0.4	−12.0±1.4
1000 hours	83	15.8±0.4	−7.1±0.4	−30.9±1.7	92	20.2±0.4	−2.4±0.3	−10.6±1.5
1500 hours	83	17.2±0.3	−4.5±0.3	−20.6±1.4	92	19.9±0.4	−1.4±0.4	−6.1±1.6
1700 hours	83	15.6±0.3	−5.9±0.3	−27.4±1.4	92	19.9±0.5	−1.2±0.4	−5.3±1.6
Mean diurnal	83	17.0±0.3	−5.7±0.3	−24.9±1.2	92	20.4±0.4	−2.0±0.3	−8.5±1.3

Abbreviations: BBFC, fixed-combination brinzolamide 1%/brimonidine 0.2% IOP, intraocular pressure; PGA, prostaglandin analog; SE, standard error.
