# Supplementary material for: Ocular hypotensive effect of fixed-combination brinzolamide/brimonidine adjunctive to a prostaglandin analog: a randomized clinical trial
Source: Eye (Lond). 2016 Jul 1;30(10):1343–50. doi: 10.1038/eye.2016.126 (PMC5129854; doi:10.1038/eye.2016.126)
Supplement: Supplementary Information [file eye2016126x1.doc]

**Table S1. Adverse Events**

| **Parameter, n (%)** | **BBFC+PGA**  **(n=93)** | **Vehicle+PGA**  **(n=95)** |
| --- | --- | --- |
| Total AEs | 33 (35.5) | 20 (21.1) |
| Serious AEs | 1 (1.1)^a^ | 0 |
| Discontinuation due to an AE | 10 (10.8) | 1 (1.1) |
| Treatment-related AEs (incidence ≥1%) | 23 (24.7) | 8 (8.4) |
| Blurred vision | 9 (9.7) | 6 (6.3) |
| Pruritus | 6 (6.5) | 0 |
| Eye irritation | 5 (5.4) | 1 (1.1) |
| Ocular hyperemia | 5 (5.4) | 1 (1.1) |
| Eyelid edema | 3 (3.2) | 0 |
| Foreign body sensation | 3 (3.2) | 0 |
| Eyelid margin crusting | 2 (2.2) | 1 (1.1) |
| Conjunctival follicles | 2 (2.2) | 0 |
| Conjunctival hyperemia | 2 (2.2) | 0 |
| Eye discharge | 2 (2.2) | 0 |
| Punctate keratitis | 2 (2.2) | 0 |
| Reduced visual acuity | 2 (2.2) | 0 |
| Dysgeusia | 2 (2.2) | 0 |

AE=adverse event; BBFC=fixed-combination brinzolamide 1%/brimonidine 0.2%; PGA=prostaglandin analog.

^a^Patient experienced 1 event each of hypoglycemia and metastatic malignant melanoma; both were unrelated to treatment.

**eFigure 1. Patient disposition.**


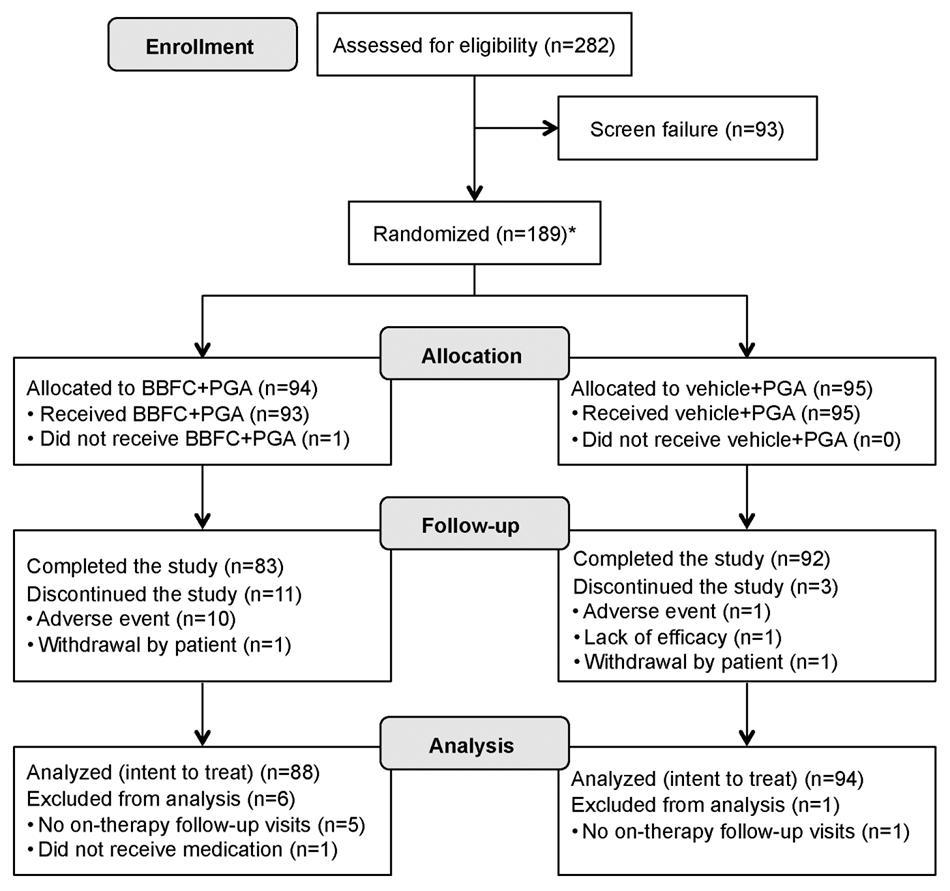


BBFC=fixed-combination brinzolamide 1%/brimonidine 0.2%; PGA=prostaglandin analog. *1 patient randomized to BBFC+PGA did not receive study medication.
